# Early implementation and contextual determinants of the human papillomavirus vaccine rollout and uptake in Nigeria: a mixed-methods study

**DOI:** 10.3389/fpubh.2026.1834756

**Published:** 2026-06-02

**Authors:** Gbemisola T. Olubayo, Ayodamola Bakare, Julius Salako, Olumide E. Olufayo, Ayobami A. Bakare

**Affiliations:** 1 Department of Community Medicine, University College Hospital, Ibadan, Nigeria; 2 Oxygen for Life Initiative, Ibadan, Nigeria; 3 Department of Global Public Health, Karolinska Institutet (KI), Solna, Sweden

**Keywords:** healthcare workers, HPV vaccination, Nigeria, vaccine availability, vaccine uptake

## Abstract

**Background:**

In 2023, Nigeria introduced the Human Papillomavirus (HPV) vaccine into routine immunization to reduce the high burden of cervical cancer. However, limited evidence exists regarding early vaccine availability, uptake, and the contextual factors influencing implementation across health facilities.

**Methods:**

This study employed a sequential explanatory mixed-methods design (quan + QUAL) across five Nigerian states (Oyo, Jigawa, Kano, Lagos, and Rivers). Data collectors conducted monthly visits to assess HPV vaccine availability and reviewed the facility register to assess HPV vaccine uptake. Subsequently, in-depth interviews were conducted with healthcare workers to explore contextual determinants of vaccine delivery and acceptance. Qualitative data were analyzed using reflexive thematic analysis and reported in accordance with COREQ guidelines.

**Result:**

Overall, 13 of the 52 (25%) facilities included in this study did not record a single HPV vaccine availability and uptake throughout the entire 4 months period. HPV vaccine availability varied substantially across states and facilities, with only 8/17 facilities in Oyo reporting availability compared to 6/11 in Jigawa across all 4 months. Uptake also varied widely, ranging from 0 to 308 doses per facility over the 4 months, with higher uptake observed in parts of Rivers State. Qualitative themes supported these findings, demonstrating that HPV vaccine uptake evolved as communities increasingly understood its relevance through local narratives, trusted health worker endorsement, and peer influence. Despite a structured rollout, frontline preparedness and vaccine uptake were shaped by variability in training, outreach-driven delivery, trust in healthcare workers and community influencers, as well as sociocultural barriers, misconceptions, and household power dynamics.

**Conclusion:**

Overall, our study reinforces that improving HPV vaccine coverage requires more than expanding stock; it demands reliable access, responsive service delivery models, and socially grounded communication strategies that foster community legitimacy. Addressing both structural constraints and contextual determinants will be essential to prevent HPV vaccination from remaining concentrated in a few facilities and instead embed it as a routine, equitable public health intervention.

## 1 Introduction

Human papillomavirus (HPV) is one of the most common sexually transmitted infections, affecting most sexually active individuals at some point (1). While many infections clear spontaneously, persistent high-risk strains can lead to genital warts and cancers, particularly cervical cancer, the fourth most common cancer among women globally (1). In 2022, about 660,000 new cervical cancer cases and 350,000 related deaths were recorded globally (1), with approximately 95% occurring in low- and middle-income countries, especially in sub-Saharan Africa (2).

Nigeria carries a significant portion of this burden. In 2020, the country reported 12,075 new cervical cancer cases and 7,968 deaths (3), ranking fifth globally (3). Among girls aged 9–20 years in Northern Nigeria, the prevalence of HPV infection has been reported at 13.2% (4). Without preventive measures such as HPV vaccination, this burden is likely to continue increasing.

Although HPV vaccines are effective and recommended for cervical cancer prevention, uptake in Nigeria remains low (5–8). A key contributor is persistent vaccine hesitancy, lack of awareness and non-availability in the national routine immunization scheme (6, 8). In October 2023, Nigeria introduced the single-dose HPV vaccine into the routine immunization schedule, beginning the first phase of rollout among girls aged 9–14 years across 16 states, with plans to reach over 17 million girls nationwide within 2 years, representing the largest single-dose HPV vaccination campaign to date (9). Before the roll-out exercise, HPV vaccination has not yet been fully integrated into routine immunization schedules, and comprehensive national data on vaccination coverage and determinants of uptake among HCWs remain limited. The 2018 National HPV Immunization Program report highlighted the absence of targeted outreach strategies and noted insufficient information on provider attitudes, awareness, and practices (10). This evidence gap constrains the development of responsive and culturally appropriate vaccination strategies.

With the recent nationwide rollout of the single-dose HPV vaccination program in Nigeria, the role of healthcare workers in delivering and sustaining this initiative has become increasingly central. Understanding healthcare workers’ experiences of the roll-out and vaccine uptake is important to improve service delivery and future vaccines roll-out and integration into the national immunization schedule. Therefore, this study aims to assess HPV vaccine uptake, availability, and the contextual factors influencing vaccination among healthcare workers in Nigeria using a sequential explanatory mixed-methods design.

## 2 Materials and methods

### 2.1 Study design

This study employed a sequential explanatory mixed-methods design, quantitative followed by qualitative (quan+QUAL). In the first phase, quantitative data were collected to determine HPV vaccine availability and uptake across selected primary healthcare facilities. In the second phase, qualitative data were collected to obtain healthcare workers’ experience of vaccine rollout, explore the contextual factors influencing vaccination uptake and explain findings from the quantitative phase. In this design, the qualitative findings were used to help interpret and provide deeper insight into the quantitative results.

The qualitative component used semi-structured interviews and was analyzed using reflexive thematic analysis (11). This approach was chosen because it allows for an inductive and interpretive understanding of participants’ experiences, perceptions, and meanings. Both quantitative and qualitative data were collected between January 2025 and April 2025. The study adhered to the Consolidated Criteria for Reporting Qualitative Research (COREQ) to ensure transparency and rigor (12).

### 2.2 Study setting

The study was conducted in five Nigerian states representing diverse geopolitical and health system contexts: Kano, Jigawa, Lagos, Oyo, and Rivers States. These states were purposively selected to reflect variation in population size, health infrastructure, and immunization program implementation across Northern and Southern Nigeria. Nigeria introduced the HPV vaccine nationwide using a phased multi-age cohort (MAC) approach, administering a single-dose quadrivalent HPV recombinant vaccine. Phase 1 of the rollout occurred in October 2023 and covered 16 states and the Federal Capital Territory, while Phase 2 took place in May 2024 and involved the remaining 21 states (13, 14). Lagos, Kano and Jigawa states were in phase 1, and Oyo and River states were in phase 2 (See Appendix 1). Routine immunization coverage differs across the five selected states. According to the Multiple Indicator Cluster Survey (MICS) & National Immunization Coverage Survey (NICS) (15), it was recorded that 72.2% of children aged 0–23 months have received all the basic vaccinations (BCG, OPV3, DTP3 and Measles 1) in Lagos state compared to 43.2, 32.5, 39.4 and 62.7% in Jigawa, Kano, Oyo and River states, respectively.

### 2.3 Sampling procedure

A multistage sampling approach was utilized. In the first stage, states and Local Government Areas (LGAs) were purposively selected to reflect Nigeria’s geographic, economic, and cultural diversity, as well as the presence of ongoing studies. Within each state, we selected between one and five Local Government Areas (LGAs) based on accessibility and feasibility of data collection, resulting in a total of 18 LGAs across the five states: Lagos (*n* = 1), Oyo (*n* = 5), Kano (*n* = 4), Jigawa (*n* = 5), and Rivers (*n* = 3). In the second stage, health facilities within the selected LGAs were conveniently sampled based on accessibility and approval from facility administrators. A total of 52 Primary Health Care (PHC) facilities were included, comprising 17 facilities in Oyo, 11 in Jigawa, 8 in Kano, 10 in Lagos, and 6 in Rivers State. In the third stage, study participants were recruited for the Qualitative phase. Healthcare workers were enrolled using convenience sampling within the selected facilities.

Based on the findings from the quantitative analysis, we conducted semi-structured interviews with healthcare workers from purposively selected facilities to explore observed trends in vaccine availability and uptake. We included different facilities with poor or good HPV vaccine availability, those who reported no or some level of HPV vaccine uptake. In the selected facilities, we recruited 44 participants based on their professional role and experience with the HPV vaccination rollout (See Appendix 2).

### 2.4 Data collection

Data collectors conducted one visit per facility each month over a four-month period (January to April 2025) to assess HPV vaccine availability and uptake. Each monthly visit was conducted after the end of the reference month; thus, January data were collected in February, February data in March, March data in April, and April data in May. This approach ensured that each month had been fully completed before data extraction and counting were undertaken. HPV vaccine availability was defined as the presence of at least one dose of the vaccine at the health facility at the time of the visit. HPV vaccine uptake was assessed by reviewing facility immunization registers and defined as the number of HPV vaccine doses administered during each completed month of the study period.

For the qualitative, a total of 44 semi-structured interviews were conducted with healthcare workers and immunization officers, directly involved in HPV vaccine implementation or communication activities. Interviews were conducted face-to-face at participants’ facilities based on their preference and convenience. Interviews were conducted in English in Lagos, Oyo, and Rivers States, and in Hausa in Jigawa and Kano States. All interviews were audio-recorded with participants’ consent and subsequently transcribed verbatim. Data collection was carried out by trained research assistants experienced in qualitative interviewing and fluent in English and the relevant local dialects. In Oyo State, interviews were conducted by OG and OO, both of whom have postgraduate training in public health. Interviews in the other states were conducted by trained BSc-level research nurses following the same standardized protocol. None of the research assistants had prior personal relationships with participants. No repeat interviews were conducted. The interview guide covered: awareness and knowledge of HPV and its vaccine; experiences with vaccine rollout; willingness to vaccinate; confidence discussing the vaccine; and perceived barriers, facilitators, and recommendations (see Appendix 3).

### 2.5 Data analysis

For the quantitative data, descriptive statistics (frequencies and percentages) were used to summarize vaccine availability and uptake patterns across facilities and states. Results were presented in tables and charts. Analysis focused on identifying geographic and facility-level variations.

For qualitative analysis, transcripts were independently reviewed by GO and AAB for familiarization. Data were analyzed using reflexive thematic analysis (11), following the steps of familiarization. Thereafter, GO independently did the initial coding, which was refined into categories and themes through iterative discussions with AAB. Analysis remained reflexive and iterative, focusing on underlying meanings and processes shaping vaccination practices.

### 2.6 Reflexivity

The research team acknowledged that personal backgrounds, professional training, and positionalities could shape interpretation. AAB, a male community health physician, brought clinical, community-focused and system-level perspectives. GO, a female public health researcher, added non-clinical perspectives. Continuous reflexive discussions were held within the core and wider research team to minimize interpretive bias.

### 2.7 Trustworthiness

We included participants across multiple states and ensured interviews were conducted in participants’ preferred languages. Triangulation occurred across quantitative and qualitative data and across researchers with varied disciplinary backgrounds (public health data analysis, community health, and public health research). Findings were reviewed and discussed by the wider study team.

## 3 Results

### 3.1 Availability and uptake of HPV vaccine

Table 1 shows the availability and uptake of Human Papillomavirus (HPV) vaccines across selected health facilities and outreach sites in Oyo, Jigawa, Kano, Lagos, and Rivers States between January and April 2025. Overall, HPV vaccine availability varied considerably across states, facilities, and months, with uptake generally occurring only in facilities where the vaccine was available.

**Table 1:** Availability and uptake of HPV vaccines in selected facilities in Oyo, Jigawa, Kano, Lagos and Rivers states.

Vaccines	HPV availability (Total doses given)									
Oyo state	Jan 2025		Feb 2025		Mar 2025		Apr 2025		Total	
Availability	Yes–Yes/No (doses administered)		Yes–Yes/No (doses administered)		Yes–Yes/No (doses administered)		Yes–Yes/No (doses administered)			
Outlets	F	O	F	O	F	O	F	O	F	O
Facility 1	Yes (0)	Yes (0)	No (0)	No (0)	No (0)	No (0)	No (0)	No (0)	0	
Facility 2	No (0)	No (0)	No (0)	No (0)	No (0)	No (0)	No (0)	No (0)	0	
Facility 3	Yes (30)	Yes (0)	Yes (0)	Yes (0)	Yes (0)	Yes (0)	Yes (4)	Yes (0)	34	
Facility 4	No (0)	No (0)	Yes (92)	Yes (0)	Yes (61)	Yes (0)	Yes (0)	Yes (0)	153	
Facility 5	No (0)	No (0)	Yes (8)	Yes (0)	Yes (0)	Yes (0)	Yes (0)	Yes (7)	8	7
Facility 6	No (0)	No (0)	Yes (61)	Yes (0)	Yes (63)	Yes (0)	Yes (61)	Yes (0)	185	
Facility 7	No (0)	No (0)	No (0)	No (0)	No (0)	No (0)	No (0)	No (0)	0	
Facility 8	No (0)	No (0)	No (0)	No (0)	No (0)	No (0)	No (0)	No (0)	0	
Facility 9	Yes (13)	Yes (7)	Yes (46)	Yes (0)	Yes (35)	Yes (0)	Yes (10)	Yes (0)	111	
Facility 10	No (0)	No (0)	No (0)	No (0)	No (0)	No (0)	No (0)	No (0)	0	
Facility 11	No (0)	No (0)	Yes (14)	Yes (0)	Yes (0)	No (0)	Yes (14)	Yes (0)	28	
Facility 12	Yes (10)	Yes (0)	Yes (9)	Yes (0)	Yes (11)	Yes (0)	Yes (19)	Yes (0)	49	
Facility 13	No (0)	No (0)	No (0)	No (0)	No (0)	No (0)	No (0)	No (0)	0	
Facility 14	No (0)	No (0)	Yes (8)	Yes (0)	Yes (13)	Yes (0)	Yes (18)	Yes (0)	39	
Facility 15	Yes (0)	Yes (0)	Yes (0)	Yes (0)	Yes (0)	Yes (0)	Yes (0)	Yes (0)	0	
Facility 16	Yes (8)	Yes (0)	Yes (9)	Yes (11)	Yes (0)	Yes (0)	No (0)	No (0)	28	
Facility 17	No (0)	No (0)	No (0)	No (0)	No (0)	No (0)	No (0)	No (0)	0	

Vaccines	HPV Availability (Total doses given)									
In Jigawa state	Jan 2025		Feb 2025		Mar 2025		Apr 2025		Total	
Availability	Yes–Yes/No (doses administered)		Yes–Yes/No (doses administered)		Yes–Yes/No (doses administered)		Yes–Yes/No (doses administered)			
Outlets	F	O	F	O	F	O	F	O	F	O
Facility 1	Yes (0)	Yes (0)	Yes (2)	Yes (0)	Yes (0)	Yes (0)	Yes (3)	Yes (0)	5	
Facility 2	Yes (0)	Yes (0)	Yes (0)	Yes (0)	No (0)	No (0)	No (0)	No (0)	0	
Facility 3	Yes (0)	Yes (0)	Yes (2)	Yes (0)	Yes (0)	Yes (0)	Yes (0)	Yes (0)	2	
Facility 4	Yes (0)	Yes (0)	Yes (3)	Yes (1)	Yes (0)	Yes (0)	Yes (0)	Yes (0)	3	1
Facility 5	Yes (0)	Yes (0)	Yes (0)	Yes (0)	Yes (0)	Yes (0)	Yes (0)	Yes (0)	0	
Facility 6	Yes (4)	Yes (0)	Yes (5)	Yes (0)	No (0)	No (0)	No (0)	No (0)	9	
Facility 7	No (0)	No (0)	Yes (0)	Yes (0)	Yes (17)	Yes (0)	No (0)	No (0)	17	
Facility 8	Yes (0)	Yes (0)	Yes (0)	Yes (0)	Yes (0)	Yes (0)	Yes (0)	Yes (0)	0	
Facility 9	No (0)	No (0)	Yes (0)	Yes (0)	Yes (44)	Yes (0)	Yes (0)	Yes (0)	44	
Facility 10	Yes (0)	Yes (4)	Yes (0)	Yes (19)	Yes (0)	Yes (5)	No (0)	No (0)	0	28
Facility 11	Yes (68)	Yes (8)	Yes (13)	Yes (0)	Yes (20)	Yes (3)	Yes (30)	Yes (14)	131	25

In Kano state	Jan 2025		Feb 2025		Mar 2025		Apr 2025		Total	
Availability	Yes–Yes/No (doses administered)		Yes–Yes/No (doses administered)		Yes–Yes/No (doses administered)		Yes–Yes/No (doses administered)			
Outlets	F	O	F	O	F	O	F	O	F	O
Facility 1	Yes (0)	Yes (0)	Yes (0)	Yes (0)	Yes (0)	Yes (0)	Yes (0)	Yes (0)	0	
Facility 2	No (0)	No (0)	No (0)	No (0)	No (0)	No (0)	No (0)	No (0)	0	
Facility 3	No (0)	No (0)	No (0)	No (0)	No (0)	No (0)	No (0)	No (0)	0	
Facility 4	Yes (0)	Yes (0)	Yes (17)	Yes (0)	Yes (0)	Yes (0)	Yes (0)	Yes (0)	17	
Facility 5	No (0)	No (0)	No (0)	No (0)	No (0)	No (0)	No (0)	No (0)	0	
Facility 6	Yes (0)	Yes (30)	Yes (0)	Yes (40)	Yes (0)	Yes (50)	Yes (0)	Yes (40)		160
Facility 7	Yes (15)	Yes (0)	Yes (25)	Yes (0)	No (0)	No (0)	Yes (14)	Yes (0)	1	
Facility 8	No (0)	No (0)	No (0)	No (0)	No (0)	No (0)	No (0)	No (0)	0	

In Lagos state	Jan 2025		Feb 2025		Mar 2025		Apr 2025		Total	
Availability	Yes–Yes/No (doses administered)		Yes–Yes/No (doses administered)		Yes–Yes/No (doses administered)		Yes–Yes/No (doses administered)			
Outlets	F	O	F	O	F	O	F	O	F	O
Facility 1	No (0)	No (0)	No (0)	No (0)	Yes (2)	Yes (0)	Yes (4)	Yes (0)	6	
Facility 2	No (0)	No (0)	No (0)	No (0)	No (0)	No (0)	Yes (70)	Yes (0)	70	
Facility 3	No (0)	No (0)	No (0)	No (0)	Yes (10)	Yes (0)	Yes (30)	Yes (0)	40	
Facility 4	No (0)	No (0)	No (0)	No (0)	Yes (0)	Yes (0)	No (0)	No (0)	0	
Facility 5	Yes (0)	Yes (0)	No (0)	No (0)	No (0)	No (0)	No (0)	No (0)	0	
Facility 6	No (0)	No (0)	No (0)	No (0)	Yes (1)	Yes (0)	Yes (1)	Yes (0)	2	
Facility 7	No (0)	No (0)	No (0)	No (0)	No (0)	No (0)	No (0)	No (0)	0	
Facility 8	Yes (0)	Yes (0)	Yes (0)	Yes (0)	Yes (0)	Yes (0)	No (0)	No (0)	0	
Facility 9	No (0)	No (0)	No (0)	No (0)	Yes (5)	Yes (0)	Yes (1)	Yes (0)	6	
Facility 10	Yes (110)	Yes (0)	No (0)	No (0)	Yes (0)	Yes (0)	No (0)	No (0)	110	

Rivers state	Jan 2025		Feb 2025		Mar 2025		Apr 2025		Total	
Availability	Yes–Yes/No (doses administered)		Yes–Yes/No (doses administered)		Yes–Yes/No (doses administered)		Yes–Yes/No (doses administered)			
Outlets	F	O	F	O	F	O	F	O		
Facility 1	Yes (1)	Yes (5)	Yes (0)	Yes (5)	Yes (0)	Yes (28)	Yes (0)	Yes (0)	1	38
Facility 2	Yes (93)	Yes (0)	No (0)	No (0)	Yes (0)	Yes (0)	No (0)	No (0)	93	
Facility 3	Yes (0)	Yes (0)	Yes (0)	Yes (0)	Yes (0)	Yes (206)	Yes (0)	Yes (102)		308
Facility 4	No (0)	No (0)	No (0)	No (0)	Yes (96)	Yes (0)	Yes (178)	Yes (0)	274	
Facility 5	Yes (0)	Yes (4)	Yes (3)	Yes (1)	Yes (0)	Yes (19)	Yes (0)	Yes (44)	3	64
Facility 6	Yes (6)	Yes (0)	Yes (19)	Yes (0)	Yes (12)	Yes (2)	Yes (28)	Yes (0)	65	2

F, Fixed outlet; O, Outreach.

In Oyo State, HPV vaccine availability was inconsistent across the 17 assessed facilities, with only 8 facilities reporting both vaccine availability and administration during the study period. Overall, vaccine uptake through facility-based services was higher compared to outreach activities. Notably, only three facilities administered more than 100 doses over the four-month period, indicating that high uptake was concentrated in a small number of sites.

In Jigawa State, most facilities (6 of 11) reported consistent HPV vaccine availability throughout the four-month study period, while the remaining five facilities experienced intermittent (staggered) availability. Despite this, vaccine uptake remained low in many facilities even when vaccines were available. Only one of the 11 facilities administered up to 100 doses across both facility-based and outreach services. In contrast, the remaining facilities administered between 2 and 44 doses, with many reporting fewer than 10 doses over the four-month period, indicating generally low utilization.

In Kano State, HPV vaccine availability was limited to only a few facilities. Four of the eight facilities assessed did not have vaccines throughout the entire four-month period, while three facilities reported consistent availability across all 4 months, one facility reported availability in three of the 4 months. Of the three facilities that reported availability, the facility with the highest uptake administered 160 doses, while one did not administer a single dose despite consistent availability, indicating substantial variation in utilization. Notably, one of the facilities in Kano State reported administering HPV vaccines through outreach services during the study period.

In Lagos State, HPV vaccine availability was generally low across the assessed facilities. Only one of the 10 facilities reported vaccine availability in January and February. However, there was a slight improvement in March and April, with four facilities reporting availability in both months. In terms of uptake, only three facilities administered up to 40 doses during the four-month period, while the remaining facilities administered fewer than 10 doses or reported no doses at all, indicating persistently low utilization despite the modest improvement in availability. The facility that administered the highest number of doses was not among the four facilities with consistent vaccine availability and surprisingly, all of its doses represented the only vaccines administered across all facilities in the month of January, as all other reported doses were administered in March and April.

In Rivers State, HPV vaccine was available from January to April in four of the six facilities included. Result from the state shows a relatively higher uptake compared to other states, even though the state had the least number of facilities represented. All six facilities administered at least one dose, either through facility services or outreach services. The lowest reported value, irrespective of service site, was 39, whereas the highest was 308. Outreach activities contributed substantially to vaccine uptake in some facilities, with three facilities reporting more outreach administration than facility administration.

Overall, HPV vaccine uptake was higher in facility-based services compared to outreach services, although some outreach activities contributed significantly to vaccine delivery in Rivers and some part of Kano states. A number of facilities reported vaccine availability without corresponding uptake, suggesting possible gaps in service delivery, demand generation, or documentation.

### 3.2 Qualitative findings

#### 3.2.1 Community reinterpretation of HPV vaccine relevance

Findings revealed a gradual repositioning of community members in relation to the HPV vaccine (Table 2). At the onset of rollout, caregivers engaged the vaccine as an externally introduced intervention whose relevance to their immediate realities was unclear. Limited knowledge about HPV and cervical cancer contributed to uncertainty, and the vaccine was initially perceived as distant from everyday experience. Over time, however, this positioning shifted as HPV and cervical cancer became contextualized through relatable narratives and lived experience.

**Table 2:** Themes and codes from analysis of transcripts.

Themes	Codes
Community reinterpretation of HPV vaccine relevance	Tangible convictions: vaccine as a shield against threats Narratives from survivors Knowledge about cervical cancer and HPV risks Health workers’ self-administration and vaccinating their own children Peer-to-peer advocacy Teachers vaccinating their daughters to convince parents/students Approval from religious leaders as catalysts for acceptance
Structured rollout, fragmented preparedness	Preparedness and confidence Negotiating acceptance through social influence Community-facing delivery and engagement Dynamics
Trust as the engine of HPV vaccine uptake	Awareness shapes demand Social meaning and trust influence acceptance Gradual normalization through social proof Awareness of cervical cancer risks Survivor testimonies and community stories Teachers, peers, religious/community leaders HCWs modeling vaccine acceptance Outreach strategies bringing vaccines closer (schools, homes, markets, ceremonies) Use of simple/pidgin English for less-educated mothers
Power, morality and misinformation	Disapproval from fathers (male involvement/household decision-making) Cultural/religious resistance (Muslim communities, large churches) Stigma/privacy concerns (parents preferring private facilities in Lagos) Rumors: infertility, population control, contraceptives Misinformation from even trained HCWs “Free means unsafe” perception Lack of incentives to encourage uptake Fear of injections Restriction to girls 9–14 is seen as exclusionary
Sustaining legitimacy beyond introduction	Extend eligibility beyond 9–14 years (older adolescents, HIV-positive clients, women up to 45) Regular re-campaigns and government follow-up Incentives for HCWs and communities Strengthened sensitization through repeated, tailored outreach (mosques, schools, markets, media) Consistent training and refresher courses for HCWs Improved logistics (solar-powered storage, consistent supply chain, better documentation tools)

Healthcare workers described how the presence of cervical cancer survivors during sensitization activities altered how caregivers perceived the vaccine. Rather than receiving HPV as a technical or biomedical explanation, community members encountered it through embodied testimony.

*“…there was a day, a woman who is still on treatment (for cervical cancer) came and told the parents that they were lucky. She said she was not lucky, that is why she is bringing her child here (to receive HPV vaccine)…” (Female HCW, Rivers State)*.

Through such encounters, cervical cancer was no longer framed as a distant possibility but as a present and visible condition affecting someone within the community. This shifted caregivers from passive listeners to individuals confronted with tangible vulnerability. HPV prevention became anchored in lived consequences rather than abstract risk statistics. In this way, positionality shifted from detachment to identification. Caregivers began to interpret the vaccine not merely as a government initiative, but as a preventive measure embedded within recognizable social realities.

Repositioning also occurred through discussions around vaccine eligibility. Among the HCWs, it was reported that questions about why the vaccine targeted girls exclusively and excluded boys or older adolescents.

As one participant explained:

*“…they say, why government did not give for male child? Why only female?” (HCW, Rivers State)*.

These reactions suggest that caregivers were not only assessing vaccine safety but also evaluating its moral and social logic. The female-only framing prompted reflection on gendered responsibility, with some interpreting the policy as placing reproductive health burden primarily on girls. Such questioning did not necessarily indicate rejection; rather, it signaled an active effort to situate the vaccine within local understandings of fairness and shared responsibility. In negotiating these meanings, community members were repositioning themselves from uninformed recipients to critical interpreters of public health policy.

As HPV became contextualized through local narratives and moral dialog, its perceived importance evolved. What began as uncertainty rooted in unfamiliarity gradually shifted toward recognition of its preventive significance. Thus, the shift in positionality was not merely about increased acceptance but about reinterpreting HPV prevention as locally meaningful. This pattern was observed across all five states, although the depth and pace of reinterpretation varied. In Rivers, Oyo and parts of Jigawa and Kano, where outreach activities, school-based delivery and visible endorsement by health workers and community figures were more sustained, the shift appeared more consolidated and publicly reinforced. In contrast, in Lagos and Kano, the transition was more uneven and often dependent on individual health worker engagement or episodic sensitization efforts. In these contexts, reinterpretation occurred, but remained more fragile and situational rather than collectively embedded.

#### 3.2.2 Structured rollout, fragmented preparedness

Several HCWs described the rollout as organized and hierarchical, involving coordinated actors such as vaccinators, recorders, and community mobilisers. In these contexts, the campaign approach appeared planned and structured:

*“…ok, when the HPV vaccine was introduced from the federal government level down to the state level and to the local government level, the campaign was carried out ward by ward. Though the training took place before the rollout was held, some healthcare workers from various facilities were involved, there are also recorders, vaccinations and community mobilizers all involved in the training, the community mobilizers were responsible for announcing the campaign to be carried out.” (HCW, Jigawa State)*.

This account reflects a coordinated implementation framework in which roles were clearly defined and preparatory activities preceded vaccine deployment. However, such structured preparation was not universal. In contrast, some health workers reported receiving no hands-on training prior to vaccine introduction:

*“No, I have never had any hands-on training in HPV vaccine.” (HCW, Kano State)*.

The absence of practical training contributed to uneven preparedness across facilities. Where formal instruction was limited, health workers relied on informal learning, peer exchange, or self-directed information seeking. This variability shaped confidence levels, particularly in counseling caregivers and addressing questions about eligibility, safety, and vaccine purpose. Thus, while the rollout design appeared standardized at the policy level, frontline readiness was fragmented in practice.

*“…when it comes to explanations or giving health talk, I tried my best, I cannot rate but I will say I tried my best because I would try to read about it, know we’ll about it so that I can able to give the right information or pass good knowledge to the person so that when they go out, they will not be misinformed.” (HCW, Lagos State)*.

Health workers consistently emphasized that facility-based vaccination alone was insufficient to reach the target age group of 9–14-year-old girls, who rarely visit primary health centers independently. As a result, outreach activities in schools, churches, and community gathering points became central to implementation. One participant described the stark contrast in uptake between facility and outreach settings:

*“We get more during the outreaches… In the health center, they come in a scanty manner, in a day, sometimes you can just see like 3 but if it’s an outreach, you are sure of coming back giving up to 30 or 50… We go far (administer more vaccines) with the outreaches than facility based.” (HCW, Rivers State)*.

Despite structured planning and adaptive outreach strategies, system-level constraints limited implementation continuity in several settings. Inconsistent vaccine supply, inadequate storage infrastructure, and unreliable power sources disrupted delivery processes. A participant from Kano explained:

*“Our major problem here is, we do not have solar and enough power supply… We go to nearby secondary facility… If we are lucky we get HPV vaccine, while sometimes it’s only routine ones we get… And the facility we collect from also runs out.” (HCW, Kano State)*.

This account highlights infrastructural fragility within the cold chain system, where dependence on nearby facilities and inconsistent stock availability constrained service reliability. Such instability not only interrupted service delivery but also placed additional logistical burdens on frontline workers. Similarly, shortages of vaccination cards undermined documentation and follow-up:

*“Barriers? I think the only barriers of HPV vaccine is. Now. I think we have shortage of HPV vaccine card.” (HCW, Oyo State)*.

Documentation materials are critical for tracking uptake and ensuring continuity of care. Their absence weakened record-keeping processes and complicated follow-up mechanisms, revealing how seemingly minor material shortages can have downstream implications for program coherence.

#### 3.2.3 Trust as the engine of HPV vaccine uptake

Facilities in Jigawa, Lagos, and Oyo frequently had HPV vaccines in stock, yet months without sustained outreach or targeted sensitization often resulted in minimal recorded uptake. Parents and girls rarely presented spontaneously for vaccination. This pattern suggests that supply was necessary but insufficient; uptake depended fundamentally on relational trust and social endorsement.

One of the most powerful drivers of acceptance was the visible demonstration of confidence by healthcare workers. In settings where health workers publicly vaccinated themselves or their own children, community resistance softened noticeably. As one participant explained:

*“You know they thought they wanted to reduce the population… When they saw us taking it, I mean the health workers… as we are taking it in their presence, they know that it is real. So, they began to take it also.” (HCW, Oyo State)*.

This account illustrates how skepticism, rooted in fears of infertility or population control, was countered not by technical explanation alone but by embodied reassurance. Acceptance emerged through observation. When health workers positioned themselves as participants rather than mere messengers, confidence spread gradually within the community.

Similarly, caregivers often sought explicit personal endorsement before consenting. As one HCW noted:

*“Once I say yes, it’s okay. That is the question they will now ask me, are you advising us to take it?” (HCW, Rivers State)*.

This highlights that decision-making was relationally negotiated. Caregivers did not rely solely on institutional authority but on trusted individuals whose personal assurance carried social weight.

Beyond health workers, uptake expanded through peer influence and community “mirror champions,” parents who had already vaccinated their daughters and were mobilized to share their experiences with others.

One participant described this process:

*“We invited mirror champions, those that were able to allow their daughters to have access to these vaccine… so that they can help us to relay the information as they go back, convince others to release their daughters for HPV vaccination…” (HCW, Rivers State)*.

These champions embedded the vaccine message within familiar social networks: schools, parent associations, and women’s groups. Their advocacy reframed vaccination as aligned with responsible parenting and community wellbeing. Acceptance spread through imitation and shared testimony, demonstrating that uptake was socially diffused rather than individually decided in isolation.

Uptake also increased when vaccination was physically brought into community spaces through school visits, house-to-house campaigns, and central gathering points. For 9–14-year-olds who rarely attend clinics independently, proximity enhanced not only accessibility but also legitimacy.

*“It should have been done house to house rather than having it in a facility… by so doing, we will have much turn-up of people” (HCW, Jigawa State)*.

Bringing vaccination closer to families reinforced perceptions of inclusivity and community ownership. Trust was strengthened when the intervention was integrated into familiar environments rather than confined to clinical settings.

#### 3.2.4 Power, morality and misinformation

Community acceptance of the HPV vaccine was strongly mediated by household authority structures, cultural interpretations of morality, and the credibility of information sources. Based on the report from the healthcare workers, fathers functioned as primary decision-makers in their household, limiting mothers’ ability to consent independently. Cultural and religious frameworks further shaped perceptions of necessity, particularly where vaccination was interpreted as unnecessary for “morally upright” girls or as indirectly endorsing sexual activity.

*… even with the incentive there are some men that still do not take the vaccine for their children, some do chase us out from their household, there is a man that said why cannot we give them the money alone, so we have to harm their child before giving them money, in fact, a lot of bitter words (HCW, Jigawa State)*.

In some urban contexts, such as Lagos, stigma and concerns about social judgment led parents to seek vaccination in private facilities to avoid public scrutiny or insinuations about their daughters’ sexual behavior. Across settings, the vaccine’s free provision and its restriction to girls aged 9–14 years were also viewed with suspicion, reinforcing doubts about intent and safety.

*“…because of the fact that is just released, so, like 88 % of people believe that it is introduced into vaccines to reduce the number of Lagos state. So it is not accepted…” (HCW, Lagos State)*.

Misinformation significantly amplified these structural barriers, especially when it originated from trusted actors. Rumors linking the vaccine to infertility, population control, or covert contraception circulated widely, undermining confidence. Notably, some HCWs reported receiving discouraging guidance from trained nurses, which intensified mistrust. As one HCW explained:

*“I’ve seen a nurse who tells me that I should not allow any of my daughters to take the vaccine. That when they are not having multiple sexual partners, and they are not flirting around, what is the essence of taking the HPV vaccine? That it’s unnecessary… So, that day I felt somehow… if a nurse can be telling me that…” (HCW, Lagos State)*.

#### 3.2.5 Sustaining legitimacy beyond introduction

Although the initial HPV vaccine rollout successfully established availability, respondents consistently emphasized that long-term uptake depends on sustained institutional support. Across states, healthcare workers described training not merely as technical instruction but as essential to maintaining professional authority in environments saturated with misinformation. The ability to respond confidently to caregivers’ questions was framed as critical to preserving credibility and sustaining public trust.

*“So far we have adequate information. And I think another thing that can actually help the health worker is trainings on this, so that we would have adequate information like give to the people… Some of these people would have actually gone to read ahead, and they will now come and ask us I heard this and this, I read about this. But if we do not have that adequate information, we just say, I do not know about it, but I will find out. (HCW, Lagos State)*.

In this context, knowledge functioned as a form of institutional backing: without refresher trainings, structured sensitization campaigns, and clear follow-up from the government, health workers felt exposed to doubt and less equipped to counter rumors. Beyond training, respondents identified structural boundaries that risked undermining program legitimacy. Several healthcare workers advocated expanding eligibility to older adolescents, young adults, and males, arguing that restricting the vaccine to girls aged 9–14 generated perceptions of exclusion and inequity. As one respondent expressed:

*“We that we are old age people, you do not give us, it is only children, so I’m not happy about it. Because we use our money to get it, so they only do it for between 9 and 14, and we, we are the ones that are supposed to get it.” (HCW, Oyo State)*.

Participants framed such limitations not only as logistical constraints but as moral tensions within communities that value collective protection. Additionally, practical challenges, including irregular re-campaigning, inadequate cold-chain infrastructure, stock-sharing between facilities, insufficient HPV cards, and limited incentives for mobilisers, were repeatedly highlighted as threats to continuity. Together, these findings indicate that successful introduction alone is insufficient; sustained uptake depends on reinforcing institutional credibility, addressing perceived inequities, and ensuring systemic reliability over time.

## 4 Discussion

This mixed-methods study examined contextual determinants of the Human Papillomavirus vaccine rollout and uptake in Nigeria. Our study shows HPV vaccine availability was reported in several facilities, uptake was uneven and highly concentrated in a small number of outlets, with Rivers states showing comparatively more sustained and distributed administration, while other states exhibited sporadic or facility-clustered delivery despite stock presence. Our quantitative data revealed many facilities with vaccines in stock reporting no doses given; this was supported by our qualitative findings, which showed that caregivers did not act on availability alone because their decisions were shaped by trust, understanding, and meaning attached to the vaccine. These findings align with reports from past studies which showed that low HPV vaccine coverage is linked with limited knowledge and awareness about the vaccine’s safety and purpose (16). A study from Kisumu County in Kenya reported that most parents had limited awareness and misconceptions about HPV, and that these concerns influenced decisions about whether their daughters should receive the vaccine, highlighting the key role of communication and trust in shaping uptake (17). In contexts like ours, where families and communities interpret vaccines through social and cultural lenses, availability must be paired with credible, community-aligned messaging to improve uptake.

In our study, the pattern of episodic rather than steady uptake observed in the quantitative data, sharp rises during certain months and near-zero activity at others, became clearer when viewed alongside our qualitative findings. Health workers described their vaccination efforts as tied to outreach events in schools or communities rather than routine service delivery. This resonates with evidence from studies in Uganda showing that uptake was higher when vaccination services were delivered through outreach clinics, when adolescents had multiple access points to receive the vaccine (18). In our setting, outreach strategies led to peaks in uptake by bringing the vaccine closer to the target age group. This supports the idea that HPV vaccination programs require delivery models that align with the lived realities of adolescents, who rarely access health facilities routinely (17). However, outreach activities are resource-intensive, requiring additional staffing, transport, logistics, and mobilization costs, which may not be sustainably available in the long term. While outreach can catalyze initial demand and expand coverage, over-reliance on campaign-style delivery risks making uptake episodic rather than routine. Strengthening facility-based delivery systems is therefore essential to institutionalize HPV vaccination within standard adolescent health services. Integrating vaccination into routine care, improving school–facility linkages, and ensuring consistent availability at primary health centers may offer a more sustainable pathway for maintaining uptake beyond periodic outreach efforts.

Another clear pattern in the data was the variation in uptake between facilities within the same LGA, where a small number of facilities accounted for most of the doses administered. While qualitative insights suggest that interpersonal dynamics, such as active local champions, health workers who visibly endorsed the vaccine, teachers who encouraged parents, or community members who shared personal stories of cancer, played an important role in generating demand. Similarly, findings from Nigeria show that health worker recommendations, leadership from trusted community members and credible local voices can significantly influence vaccine decisions (19, 20).

In this study, we compare RI and HPV vaccine availability and uptake, and we observed patterns that suggest important differences between the availability and uptake of HPV vaccines compared with routine immunization (RI) vaccines (see Appendix 4). Our findings show that both HPV and routine immunization (RI) vaccines were inconsistent in monthly availability across facilities. This suggests possible supply chain or distribution challenges affecting vaccine delivery in general. Similar supply-side constraints have been reported in sub-Saharan Africa, where gaps in cold-chain capacity, storage infrastructure, and forecasting systems can lead to vaccine stock-outs and uneven distribution across facilities (21).

However, RI vaccines demonstrated more consistent monthly uptake compared to HPV vaccines. This may be due to long-standing integration of RI into the national immunization program and the established monitoring structure. In contrast, even when HPV vaccines were available, uptake remained low, indicating that availability alone may not be sufficient to drive the uptake.

This low demand may be related to limited awareness about HPV and its vaccine, perceptions about the vaccines or poor community mobilization compared to RI services. Evidence from Nigeria shows that awareness of HPV and its vaccine remains low in many settings, with knowledge gaps and misconceptions significantly influencing acceptance and uptake (22). Additionally, studies have demonstrated that higher awareness is strongly associated with increased likelihood of vaccine uptake, highlighting the critical role of health education and community engagement (23, 24). These findings suggest that, beyond improving the supply chain, there is a need for stronger community sensitization and awareness, as well as better integration of HPV vaccines into routine immunization services to improve uptake.

Mixed feelings in communities about the strict eligibility criteria (targeting only girls aged 9–14) emerged as an important explanatory theme for the observed uptake patterns. Health workers reported that some parents expressed confusion or resentment regarding both the age and gender restrictions, questioning why older girls or boys were excluded from vaccination. According to these accounts, the policy was sometimes perceived as arbitrary or insufficiently explained, particularly in households with male children or daughters outside the eligible age bracket. These concerns, as described by health workers, may have discouraged engagement in certain settings and thereby contributed to lower vaccine uptake. This aligns with broader debates in the literature about gendered perceptions of HPV vaccination and emerging evidence that HPV vaccines can also protect boys and older age groups, which has led some programs to move toward gender-neutral recommendations (25). While our study did not directly measure male vaccination, health care workers described community frustration about the exclusion of boys and older age groups from eligibility. Such reported concerns indicate that rigid policy boundaries may inadvertently undermine community buy-in, especially in contexts where the scientific rationale for targeted vaccination is not clearly explained. Studies have shown that gender-restricted vaccination policies can shape community perceptions of fairness and influence vaccine acceptability, particularly when the rationale for prioritizing girls is not clearly communicated (26–28).

Our findings do not suggest that a phased roll-out affects HPV vaccine availability and uptake, but rather that contextual factors at the state-level appear to exert greater influence. The increased availability observed at Jigawa is not surprising given the political commitment to improve vaccine uptake, which has led to improvement in routine immunization from 7% in 2016 to 49% in 2024 (29). Findings from the qualitative data also support this, as participants from Jigawa reported receiving adequate training on HPV vaccine, unlike in Kano state, where participants described limited training exposure.

Additionally, structural health system challenges also emerged as important. Discrepancies in reported vaccination numbers across facilities may partly reflect documentation gaps rather than the true absence of vaccination activity. In the qualitative interviews, some health care workers reported that doses administered during outreach activities were not always recorded in facility registers, suggesting that routine reporting systems may not fully capture all vaccinations delivered. These documentation gaps were accompanied by broader operational constraints described by participants, including weak cold-chain capacity, informal stock sharing between facilities, and inconsistent record-keeping practices. Such operational challenges matter because they affect the reliability and visibility of vaccination services and may ultimately undermine caregiver confidence in the program. Previous studies have similarly shown that weaknesses in supply chains, logistics, and communication systems can undermine vaccination efforts globally (18, 30, 31).

Our study underscores that a dynamic interplay among supply, access, and demand shapes HPV vaccine uptake. Across the 52 facilities reviewed, only a small proportion reported consistent vaccine availability, and among those, sustained uptake was concentrated in a limited number of sites. Facilities in Rivers, Jigawa, and Oyo generally reported more stable access to HPV vaccine stock. While facilities in Lagos and Kano, as seen in the quantitative findings, had no vaccine stock, and even when stock was available, uptake was minimal. Qualitative findings suggest that these differences were linked to operational factors, including more consistent vaccine distribution from state cold stores, better coordination between facilities and immunization hubs, and the integration of HPV vaccination into existing outreach and routine immunization activities in higher-performing states. In contrast, participants in lower-performing states described challenges including limited cold-chain capacity, reliance on informal stock sharing between facilities, and irregular supply from central stores, which constrained their ability to maintain continuous vaccination services. These findings highlight that reliable vaccine supply and distribution systems are foundational for coverage, as demand generation efforts cannot translate into vaccination when stock is unavailable. Similar evidence from low- and middle-income countries shows that supply chain reliability, cold-chain infrastructure, and integration with routine immunization platforms are critical determinants of HPV vaccine program performance and coverage outcomes (17, 26–28).

Moreover, strengthening HPV vaccination programs in Nigeria and similar contexts will require strategies that go beyond stock availability to tackle the social dimensions of vaccine acceptance. This includes partnering with local leaders, crafting culturally responsive communication, and expanding community outreach to build shared understanding of why the vaccine matters. Research suggests that such combined approaches, linking awareness generation with trusted messengers and reliable service delivery, can improve uptake and sustain it over time (32).

In summary, our findings add to a growing body of evidence showing that health interventions succeed not only when they are physically accessible, but when they are socially legitimate and meaningfully understood by the communities they are meant to serve.

### 4.1 Strengths and limitations

A key strength of this study lies in its mixed-methods approach, which allowed triangulation between facility records and HCW accounts, offering both breadth and depth. Firstly, the inclusion of diverse states supports comparative insights across different implementation contexts. However, the convenience sampling of facilities and participants may introduce bias and limit representativeness of the sample, thereby restricting the generalizability of the findings. In addition, the use of monthly visits provided standardized time points for assessment. However, availability measured at a single visit may not fully reflect stock fluctuations that occurred throughout the preceding month. A further limitation is that, incomplete or inconsistent facility registers may also affect the accuracy of the quantitative data. Similarly, while these registers are routinely used for service delivery monitoring, no independent verification (e.g., cross-checking with stock records or immunization cards) was conducted. As such, the findings may be subject to data quality limitations, including potential inaccuracies or incomplete recording.

Furthermore, due to the unavailability of reliable denominator data on the eligible target population (girls aged 9–14 years) within facility catchment areas, HPV vaccine uptake was reported as the absolute number of doses administered per facility per month, and so, we could not calculate vaccine coverage and we also we did not conduct any inferential statistical; therefore, comparisons between states and facilities are descriptive in nature and should be interpreted with caution.

Finally, the qualitative component focused solely on healthcare workers and did not incorporate caregiver perspectives, which may have constrained understanding of household decision dynamics. Nonetheless, the convergence of quantitative and qualitative findings strengthens confidence in interpretation.

## 5 Conclusion

This mixed-methods study highlights that improving HPV vaccine coverage requires more than expanding stock; it demands reliable access, responsive service delivery models, and socially grounded communication strategies that foster community legitimacy. Outreach efforts boosted uptake temporarily, yet are resource-intensive and unsustainable without strengthened facility-based delivery, for example, through integration of HPV vaccination into routine adolescent health services and scheduled immunization sessions at primary health care facilities. Our findings underscore that scaling HPV vaccination requires integrated strategies: ensuring reliable stock and access, institutionalizing routine delivery within primary health care services, and deploying culturally responsive communication and trusted local advocacy. To improve HPV vaccine uptake, integration of HPV vaccination into school health programs and adolescent health platforms is recommended to enhance reach among eligible girls. In addition, targeted training for community health workers should address common misconceptions, particularly concerns around infertility and vaccine safety, which were frequently reported in similar settings. Development of culturally appropriate and gender-sensitive communication materials is also needed to address misinformation and improve community trust. Furthermore, strengthening outreach services through scheduled community-based vaccination days may help improve access in underserved areas.

## Data Availability

The raw data supporting the conclusions of this article will be made available by the authors, without undue reservation.

## References

[1] WHO. *Cervical Cancer*. Geneva: WHO. (2025). Available online at: https://www.who.int/news-room/fact-sheets/detail/cervical-cancer (Accessed November 6, 2025).

[2] WHO. *Human Papillomavirus and Cancer*. Geneva: WHO. (2025). Available online at: https://www.who.int/news-room/fact-sheets/detail/human-papilloma-virus-and-cancer (Accessed November 6, 2025).

[3] Okolie EA Nwadike BI. Spotlight on human papillomavirus vaccination coverage: is Nigeria making any progress? JCO Glob Oncol. (2023):e2300088. doi: 10.1200/GO.23.00088 37319397 PMC10497273

[4] Cosmas NT Nimzing L Egah D Famooto A Adebamowo SN Adebamowo CA. Prevalence of vaginal HPV infection among adolescent and early adult girls in Jos, north-Central Nigeria. BMC Infect Dis. (2022) 22:340. doi: 10.1186/s12879-022-07215-7, 35382756 PMC8985253

[5] Perlman S Wamai RG Bain PA Welty T Welty E Ogembo JG. Knowledge and awareness of HPV vaccine and acceptability to vaccinate in sub-Saharan Africa: a systematic review. PLoS One. (2014) 9:e90912. doi: 10.1371/journal.pone.0090912, 24618636 PMC3949716

[6] Ojule IN Anika IE. Human papilloma virus transmission: knowledge and uptake of HPV vaccine among in-school adolescent girls in south-South Nigeria. Int J Trop Dis Health. (2020) 1:25–37. doi: 10.9734/ijtdh/2020/v41i830308

[7] Adepoju P. Nigeria targets almost 8 million girls with HPV vaccine. Lancet. (2023) 402:1612. doi: 10.1016/S0140-6736(23)02450-9, 37926106

[8] Elebiyo OT. Knowledge, attitude, and uptake of human papilloma virus (HPV) vaccine among parents of adolescents attending outpatient clinic at the University of Benin Teaching Hospital, Nigeria. Afr J Reprod Health. (2023) 27:108–17. doi: 10.29063/ajrh2023/v27i3.12, 37584977

[9] International Vaccine Access Center. *One Dose at a Time: Mobilizing to Eliminate Cervical Cancer in Nigeria*. International Vaccine Access Center. (2024). Available online at: https://publichealth.jhu.edu/ivac/2024/one-dose-at-a-time-mobilizing-to-eliminate-cervical-cancer-in-nigeria (Accessed November 7, 2025).

[10] TechNet. *Synthesis of HPV Program Learnings in Nigeria*. TechNet-21. (2026). Available online at: https://www.technet-21.org/en/resources/report/synthesis-of-hpv-program-learnings-in-nigeria (Accessed April 28, 2026).

[11] Braun V Clarke V. Reflecting on reflexive thematic analysis. Qual Res Sport, Exerc Health. (2019) 11:589–97. doi: 10.1080/2159676X.2019.1628806

[12] Tong A Sainsbury P Craig J. Consolidated criteria for reporting qualitative research (COREQ): a 32-item checklist for interviews and focus groups. Int J Qual Health Care. (2007) 19:349–57. doi: 10.1093/intqhc/mzm042, 17872937

[13] WHO. (2025). Available online at: https://www.who.int/docs/default-source/immunization/demand/summary-of-sage-vaccinehesitancy-en.pdf (Accessed November 6, 2025).

[14] Vaccines Work. *COVAX Explained*. (2025). Available online at: https://www.gavi.org/vaccineswork/covax-explained (Accessed November 6, 2025).

[15] UNICEF. *2021 Multiple Indicator Cluster Survey/ National Immunization Coverage Survey Report*. UNICEF Nigeria. (2022). Available online at: https://www.unicef.org/nigeria/reports/2021-multiple-indicator-cluster-survey-national-immunization-coverage-survey-report (Accessed April 28, 2026).

[16] Hakimi S Lami F Allahqoli L Alkatout I. Barriers to the HPV vaccination program in the eastern Mediterranean region: a narrative review. J Turk Ger Gynecol Assoc. (2023) 24:48–56. doi: 10.4274/jtgga.galenos.2022.2022-6-6, 36583290 PMC10019013

[17] Ochomo EO Tonui P Muthoka K Amboka S Itsura P Orang’o EO . Addressing HPV vaccine hesitancy: unveiling concerns and building trust perspectives of adolescent girls and parents in Kisumu County, Kenya. Ecancermedicalscience. (2024) 18:1735. doi: 10.3332/ecancer.2024.1735, 39421184 PMC11484676

[18] Nabirye J Okwi LA Nuwematsiko R Kiwanuka G Muneza F Kamya C . Health system factors influencing uptake of human papilloma virus (HPV) vaccine among adolescent girls 9-15 years in Mbale District, Uganda. BMC Public Health. (2020) 20:171. doi: 10.1186/s12889-020-8302-z, 32019543 PMC7001317

[19] Balogun FM Omotade OO. Facilitators and barriers of healthcare workers’ recommendation of HPV vaccine for adolescents in Nigeria: views through the lens of theoretical domains framework. BMC Health Serv Res. (2022) 22:824. doi: 10.1186/s12913-022-08224-7, 35752809 PMC9233785

[20] Bamogo A Wapmuk AE Osunsanmi SP Kpokiri EE Gbaja-Biamila TA Iwelunmor J . Community engagement for HPV vaccination in Nigeria. Sex Transm Infect. (2025) 101:344–5. doi: 10.1136/sextrans-2025-056496, 40295101 PMC12276920

[21] Amponsah-Dacosta E Kagina BM Olivier J. Health systems constraints and facilitators of human papillomavirus immunization programmes in sub-Saharan Africa: a systematic review. Health Policy Plan. (2020) 35:701–17. doi: 10.1093/heapol/czaa017, 32538437 PMC7294244

[22] Ogundipe L Ojo T Oluwadare T Olayemi E Oluwafemi F Oni O . Cervical cancer screening and vaccination: knowledge, awareness, and attitude of female staff in a Nigerian university. BMC Womens Health. (2023) 23:218. doi: 10.1186/s12905-023-02345-9, 37138288 PMC10157973

[23] Okagbue HI Erekosima G Sampson S Atuhaire B Samuel O Chimezie B . Predictors of willingness of HPV vaccine uptake across eight states in Nigeria. BMC Public Health. (2025) 25:745. doi: 10.1186/s12889-025-22000-2, 39994628 PMC11849155

[24] Talabi O Gilbert H Fawzi MCS Anorlu R Randall T. Examining barriers and facilitators of HPV vaccination in Nigeria, in the context of an innovative delivery model: a mixed-methods study. BMJ Public Health. (2023) 1:e000003. doi: 10.1136/bmjph-2023-000003, 40017875 PMC11812689

[25] Johnson CK. *HPV Vaccines Prevent cancer in men as well as Women, new Research Suggests*. AP News (2024). Available online at: https://apnews.com/article/hpv-vaccine-oral-cancer-prevention-males-749bf59c10d8a5a86c71c89c50eda0cb (Accessed February 2, 2026).

[26] Fu X Guo X Lu J Zhou W Lu Y. Acceptance of human papillomavirus vaccine among boys in Asia: a narrative review. Hum Vaccin Immunother. (2024) 20:2429894. doi: 10.1080/21645515.2024.2429894, 39611606 PMC11610555

[27] Kutz J-M Rausche P Gheit T Puradiredja DI Fusco D. Barriers and facilitators of HPV vaccination in sub-saharan Africa: a systematic review. BMC Public Health. (2023) 23:974. doi: 10.1186/s12889-023-15842-1, 37237329 PMC10214362

[28] Iliassu S Mbanga C Ngenge MB Ndoula S Njoh AA Griffith BC . Perceptions of a gender-neutral approach to human papillomavirus (HPV) vaccination in Cameroon: a qualitative study. BMC Public Health. (2026) 26:730. doi: 10.1186/s12889-026-26730-9, 41724945 PMC13037256

[29] Biriowo K. *Jigawa Records 49% Immunisation Coverage Increase in five Years*. Tribune Online (2024). Available online at: https://tribuneonlineng.com/jigawa-records-49-immunisation-coverage-increase-in-five-years/ (Accessed March 9, 2026).

[30] Asaolu SO Kubura D Erekosima G Dauda M Popoola G Rauf R . Perceptions of HPV and HPV vaccines among parents and caregivers of girls aged 9–14 years in Nigeria: a qualitative study. Discov Public Health. (2025) 22:856. doi: 10.1186/s12982-025-01276-0, 41438281 PMC12719340

[31] Oku A Oyo-Ita A Glenton C Fretheim A Eteng G Ames H . Factors affecting the implementation of childhood vaccination communication strategies in Nigeria: a qualitative study. BMC Public Health. (2017) 17:200. doi: 10.1186/s12889-017-4020-6, 28202001 PMC5311723

[32] Olajire A Igbokwe U Abdulsalam AT Nwaokorie C Abraham L Ekpin V . *Edutainment for Polio Vaccination Uptake in Northern Nigeria: A Descriptive Case Study of a Theory-Driven Public Health Intervention*. (2025).

