# Supplementary material for: Early implementation and contextual determinants of the human papillomavirus vaccine rollout and uptake in Nigeria: a mixed-methods study
Source: Front Public Health. 2026 Jun 2;14:1834756. doi: 10.3389/fpubh.2026.1834756 (PMC13271000; doi:10.3389/fpubh.2026.1834756)
Supplement: Supplementary file 1 [file Table_1.docx]

**Appendix 1**

**Phases 1 and 2 of the HPV vaccine rollout**

- **Phase 1:** Commenced on October 24, 2023, covering 16 states (including Abia, Adamawa, Akwa Ibom, Bauchi, Bayelsa, Enugu, Jigawa, Kano, Kebbi, Lagos, Nasarawa, Ogun, Osun, Taraba) and the Federal Capital Territory (FCT).
- **Phase 2:** Commenced on May 27, 2024, covering the remaining 21 states (including Anambra, Borno, Cross River, Delta, Ebonyi, Edo, Ekiti, Gombe, Imo, Kaduna, Katsina, Kwara, Kogi, Niger, Ondo, Oyo, Plateau, Rivers, Sokoto, Yobe, and Zamfara).
