# Supplementary material for: Early implementation and contextual determinants of the human papillomavirus vaccine rollout and uptake in Nigeria: a mixed-methods study
Source: Front Public Health. 2026 Jun 2;14:1834756. doi: 10.3389/fpubh.2026.1834756 (PMC13271000; doi:10.3389/fpubh.2026.1834756)
Supplement: Supplementary file 2 [file Table_2.docx]

**Appendix 2**

**Socio-demographic characteristics of respondents (N = 44)**

| **Variables** | **Lagos (n= 9)** | **Oyo (n=8)** | **Rivers (n=9)** | **Kano (n=8)** | **Jigawa (n=10)** |
| --- | --- | --- | --- | --- | --- |
| **Gender** |  |  |  |  |  |
| Female | 9 | 8 | 8 | 8 | 7 |
| Male | 0 | 0 | 1 | 0 | 3 |
| **Cadre** |  |  |  |  |  |
| Bsc PH | 0 | 0 | 1 | 0 | 0 |
| CHEW (Community Health Extension Worker**)** | 2 |  | 2 | 8 | 7 |
| CHO (Community Health Officer**)** | 1 | 4 | 5 | 0 | 1 |
| EHO (Environmental Health Officer) | 0 | 2 | 0 | 0 | 1 |
| Medical Laboratory Technician | 0 | 0 | 0 | 0 | 1 |
| Nurse/midwife | 6 | 2 | 1 | 0 | 0 |
| **Level of Education** |  |  |  |  |  |
| Diploma | 2 | 3 | 0 | 5 | 8 |
| Teritary | 7 | 3 | 6 | 3 | 1 |
| Postgraduate | 0 | 2 | 3 | 0 | 1 |
| **Religion** |  |  |  |  |  |
| Christianity | 9 | 6 | 9 | 0 | 0 |
| Islam | 0 | 2 | 0 | 8 | 10 |
