# Supplementary material for: Early implementation and contextual determinants of the human papillomavirus vaccine rollout and uptake in Nigeria: a mixed-methods study
Source: Front Public Health. 2026 Jun 2;14:1834756. doi: 10.3389/fpubh.2026.1834756 (PMC13271000; doi:10.3389/fpubh.2026.1834756)
Supplement: Supplementary file 3 [file Table_3.docx]

**Appendix 3**

**HCW Interview guide for HPV vaccine study**

| **Interview demographic data collection from (parents / caregivers)** | |
| --- | --- |
| **Health facility** |  |
| **Age** |  |
| **Religion** |  |
| **Tribe** |  |
| **Occupation** |  |
| **Sex** | **□ Female**  **□ Male** |
| **Cadre of HCW** |  |
| **Education (highest level)** |  |
| **Number of years practicing/ years of experience** |  |
| **Involvement in vaccination** | **Yes**  **No** |

|  |  |  |
| --- | --- | --- |
| **1** | **Section A:**  **Demographics and Introduction** | **Can you briefly tell us about yourself.**   - ***Your current role in this facility and professional background,*** - ***how long you have been practicing in this field, and how long you have been working in this facility*** |
| **2** | **Section B:**  **Awareness and Knowledge of HPV and HPV vaccine** | **Can you tell us what you know about Human Papilloma Virus (HPV)**  **Probe for**   - **What diseases it cause, how it is contracted e.t.c** - **How can your clients/patients know if they have HPV infection** |
| **3** | **Experiences with HPV vaccine roll-out** | **Recently, the Federal Government of Nigeria introduced the HPV vaccine into the routine immunization coverage. Tell us how the roll-out was done in your state/LGA. What could have been done better in terms of HPV vaccine roll-out?**  **I want you to compare your experiences of HPV vaccine roll out in your state/LGA with other routine immunization antigens (BCG, PENTA, PCV, Measles vaccine), what is similar and different with HPV vaccine in terms of (i)supply/availability at health facility, (ii) storage and (iii) community demand.**  **What factors are responsible for these similarities and differences** |
| **4** | **SECTION C:**  **Perception of the vaccine and willingness to receive the vaccine** | **What are your personal thoughts and concerns about the HPV vaccine?**   - **How do you perceive the importance of the HPV vaccine in preventing HPV-related diseases?** - **What are your thoughts on the safety and efficacy of the HPV vaccine?** - **Have you encountered any concerns or misconceptions about the HPV vaccine among your colleagues or patients? Tell me about it, and how did you handle it** - **Since HPV roll-out in Nigeria, have you advised your relative to take it? What was their response?**   - **If no, why?** |
| **5** | **SECTION D:**  **HPV outreach programme** | **Which outreach programme do you do for HPV vaccine? How often and when last was it done? What informed the decision?**  **What have been the experiences/challenges with the HPV vaccine outreach programme** |
| **6** | **Review of quantitative result** | **We looked at HPV vaccine records for many facilities including this facility. For this facility, we found that:**   1. **HPV vaccine was available in x out y months** 2. **And number of HPV vaccine have been administered**   **What can you about this finding? Does it reflect what your experience? What is responsible for this?** |
| **7** | **SECTION E:**  **Barriers and Facilitators to HPV Vaccination and how it influences practice and recommendation** | - **What challenges do you face in administering or recommending the HPV vaccine?** - **What are the way the facility or you personally have been overcoming these challenges?** - **How can healthcare institutions better support you in promoting HPV vaccination?**   ***Probe for factors that influences their decision to recommend or not recommend the vaccine?***  ***Probe if age, religion, ethnicity affects decision etc***  ***Probe if they have ever recommended the vaccine and when last they recommended the vaccine*** |
| **8** | **SECTION F:**  **Confidence in discussing vaccination and Suggestions for improvement** | - **How can you describe your ability to discuss HPV vaccine topic with clients/patients**   ***Probe if they have received any training or resources to support HPV vaccination discussions with patients ? ask about the training (where, when, what was discussed)***   - **What recommendations do you have for improving HPV vaccination rates among eligible adolescents?** |
| **9** |  | **Any other comments** |
