# Supplementary material for: Early implementation and contextual determinants of the human papillomavirus vaccine rollout and uptake in Nigeria: a mixed-methods study
Source: Front Public Health. 2026 Jun 2;14:1834756. doi: 10.3389/fpubh.2026.1834756 (PMC13271000; doi:10.3389/fpubh.2026.1834756)
Supplement: Supplementary file 4 [file Table_4.docx]

**Appendix 4**

Availability and uptake of other RI vaccines in selected facilities in Oyo, Jigawa, Kano, Lagos and Rivers states

**BCG vaccines administered by facility in last three available months**

| **Vaccines** | **HPV (Total doses given)** | | |  |  |
| --- | --- | --- | --- | --- | --- |
| **Facility** | **Month 1** | **Month 2** | **Month 3** | **Total** | **Average** |
| ***In Oyo state*** | Available (Number administered) | Available (Number administered) | Available (Number administered) |  |  |
| Facility 1 | Available (314) | Available (100) | Available (119) | 533 | 177.7 |
| Facility 2 | Available (169) | Available (129) | Available (362) | 660 | 220 |
| Facility 3 | Available (23) | Available (90) | Available (109) | 222 | 74 |
| Facility 4 | Available (159) | Available (56) | Available (109) | 324 | 108 |
| Facility 5 | Available (53) | Available (158) | - | 211 | 70.3 |
| Facility 6 | Available (60) | Available (178) | Available (108) | 346 | 115.3 |
| Facility 7 | Available (102) | Available (72) | Available (46) | 220 | 73.3 |
| Facility 8 | Available (91) | Available (57) | Available (35) | 183 | 61 |
| Facility 9 | Available (55) | Available (19) | Available (122) | 196 | 65.3 |
| Facility 10 | Available (23) | - | - | 23 | 7.7 |
| Facility 11 | Available (24) | Available (90) | Available (5) | 119 | 39.7 |
| Facility 12 | Available (157) | Available (150) | Available (165) | 472 | 157.3 |
| Facility 13 | Available (374) | Available (57) | - | 431 | 143.7 |
| Facility 14 | Available (41) | Available (29) | Available (33) | 103 | 34.3 |
| Facility 15 | Available (30) | Available (193) | Available (150) | 373 | 124.3 |
| Facility 16 | Available (36) | Available (90) | Available (100) | 226 | 75.3 |
| Facility 17 | Available (36) | Available (109) | Available (55) | 200 | 66.7 |
| ***In Jigawa state*** |  |  |  |  |  |
| Facility 1 | Available (88) | Available (181) | - | 269 | 89.7 |
| Facility 2 | Available (94) | Available (52) | Available (135) | 281 | 93.7 |
| Facility 3 | Available (31) | Available (75) | - | 106 | 35.3 |
| Facility 4 | Available (143) | Available (147) | - | 290 | 96.7 |
| Facility 5 | Available (84) | Available (119) | Available (52) | 255 | 85 |
| Facility 6 | Available (140) | Available (150) | Available (52) | 342 | 114 |
| Facility 7 | Available (123) | Available (192) | Available (231) | 546 | 182 |
| Facility 8 | Available (124) | Not Available (0) | Available (305) | 429 | 143 |
| Facility 9 | Available (36) | Available (24) | Available (133) | 193 | 64.3 |
| Facility 10 | Available (207) | Available (102) | Available (312) | 621 | 207 |
| Facility 11 | Available (50) | Available (157) | Available (76) | 283 | 94.3 |
| ***In Kano state*** |  |  |  |  |  |
| Facility 1 | Available (36) | - | - | 36 | 12 |
| Facility 2 | Available (42) | - | - | 42 | 14 |
| Facility 3 | Available (189) | Available (104) | - | 293 | 97.7 |
| Facility 4 | Available (56) | - | - | 56 | 18.7 |
| ***In Lagos state*** |  |  |  |  |  |
| Facility 1 | Available (232) | - | - | 232 | 77.3 |
| Facility 2 | Available (28) | Available (140) | Available (116) | 284 | 94.7 |
| Facility 3 | Available (43) | - | - | 43 | 14.3 |
| Facility 4 | Available (84) | Available (149) | Available (34) | 267 | 89 |
| Facility 1 | Available (190) | Available (87) | - | 277 | 92.3 |
| Facility 2 | Available (25) | - | - | 25 | 8.3 |
| Facility 3 | Available (169) | Available (184) | - | 353 | 117.7 |
| Facility 4 | Available (232) | - | - | 232 | 77.3 |
| ***In Rivers state*** |  |  |  |  |  |
| Facility 1 | Available (98) | Available (94) | - | 192 | 64 |
| Facility 2 | Available (13) | - | - | 13 | 4.3 |
| Facility 3 | Available (231) | Available (85) | - | 316 | 105.3 |
| Facility 4 | Available (55) | - | - | 55 | 18.3 |
| Facility 5 | Available (192) | - | - | 192 | 64 |

**Pentavalent vaccines administered by facility in last three available months**

| **Vaccines** | **HPV (Total doses given)** | | |  |  |
| --- | --- | --- | --- | --- | --- |
| **Facility** | **Month 1** | **Month 2** | **Month 3** | **Total** | **Average** |
| ***In Oyo state*** | Available (Number administered) | Available (Number administered) | Available (Number administered) |  |  |
| Facility 1 | Available (901) | Available (100) | Available (97) | 1,098 | 366 |
| Facility 2 | Available (216) | Available (428) | Available (559) | 1,203 | 401 |
| Facility 3 | Available (112) | Available (271) | Available (147) | 530 | 176.7 |
| Facility 4 | Available (275) | Available (61) | Available (262) | 598 | 199.3 |
| Facility 5 | Available (131) | Available (393) | - | 524 | 70.3 |
| Facility 6 | Available (200) | Available (242) | Available (243) | 685 | 228.3 |
| Facility 7 | Available (343) | Available (0) | Available (228) | 571 | 190.3 |
| Facility 8 | Available (365) | Available (150) | Available (64) | 579 | 193 |
| Facility 9 | Available (45) | Available (70) | Available (566) | 681 | 227 |
| Facility 10 | Available (68) | - | - | 68 | 22.7 |
| Facility 11 | Available (81) | Available (267) | Available (21) | 119 | 39.7 |
| Facility 12 | Available (580) | Available (150) | Available (458) | 1,188 | 396 |
| Facility 13 | Available (840) | Available (135) | - | 975 | 143.7 |
| Facility 14 | Available (118) | Available (135) | Available (76) | 329 | 109.7 |
| Facility 15 | Available (96) | Available (423) | Available (217) | 736 | 245.3 |
| Facility 16 | Available (48) | Available (173) | Available (279) | 500 | 166.7 |
| Facility 17 | Not Available (0) | Available (109) | Not Available (0) | 109 | 36.3 |
| ***In Jigawa state*** |  |  |  |  |  |
| Facility 1 | Available (166) | Available (61) | - | 227 | 75.7 |
| Facility 2 | Available (157) | Available (120) | Available (268) | 545 | 181.7 |
| Facility 3 | Available (99) | Available (176) | - | 275 | 91.7 |
| Facility 4 | Available (427) | Available (318) | - | 745 | 248.3 |
| Facility 5 | Available (115) | Available (379) | Available (57) | 551 | 183.7 |
| Facility 6 | Available (58) | Available (278) | Available (151) | 487 | 162.3 |
| Facility 7 | Available (450) | Available (372) | Not Available (0) | 822 | 274 |
| Facility 8 | Available (350) | Available (20) | Available (777) | 1,147 | 382.3 |
| Facility 9 | Available (112) | Available (68) | Available (290) | 470 | 156.7 |
| Facility 10 | Available (512) | Available (303) | Available (472) | 1,287 | 429 |
| Facility 11 | Available (48) | Available (456) | Available (219) | 723 | 241 |
| ***In Kano state*** |  |  |  |  |  |
| Facility 1 | Not Available (0) | - | - | 0 | 0 |
| Facility 2 | Available (129) | - | - | 129 | 43 |
| Facility 3 | Available (324) | Available (330) | - | 654 | 218 |
| Facility 4 | Available (98) | - | - | 98 | 32.7 |
| ***In Lagos state*** |  |  |  |  |  |
| Facility 1 | Available (666) | - | - | 666 | 222 |
| Facility 2 | Available (2) | Available (155) | Available (379) | 536 | 178.7 |
| Facility 3 | Available (97) | - | - | 97 | 14.3 |
| Facility 4 | Available (252) | Available (317) | Available (78) | 647 | 215.7 |
| Facility 5 | Available (301) | Available (150) | - | 451 | 150.3 |
| Facility 6 | Available (25) | - | - | 25 | 8.3 |
| Facility 7 | Available (277) | Available (325) | - | 602 | 200.7 |
| Facility 8 | Available (499) | - | - | 499 | 166.3 |
| ***In Rivers state*** |  |  |  |  |  |
| Facility 1 | Available (367) | Available (241) | - | 608 | 202.7 |
| Facility 2 | Available (19) | - | - | 19 | 6.3 |
| Facility 3 | Available (670) | Available (256) | - | 926 | 308.7 |
| Facility 4 | Available (163) | - | - | 163 | 54.3 |
| Facility 5 | Available (480) | - | - | 480 | 160 |

**Measles vaccines administered by facility in last three available months**

| **Vaccines** | **HPV (Total doses given)** | | |  |  |
| --- | --- | --- | --- | --- | --- |
| **Facility** | **Month 1** | **Month 2** | **Month 3** | **Total** | **Average** |
| ***In Oyo state*** | Available (Number administered) | Available (Number administered) | Available (Number administered) |  |  |
| Facility 1 | Available (921) | Available (62) | Available (190) | 1,173 | 391 |
| Facility 2 | Available (174) | Available (436) | Available (533) | 1,143 | 381 |
| Facility 3 | Available (53) | Available (169) | Available (120) | 530 | 176.7 |
| Facility 4 | Available (259) | Available (44) | Available (230) | 533 | 177.7 |
| Facility 5 | Available (85) | Available (289) | - | 374 | 124.7 |
| Facility 6 | Available (110) | Available (224) | Available (157) | 491 | 163.7 |
| Facility 7 | Available (207) | Available (84) | Available (122) | 413 | 137.7 |
| Facility 8 | Available (219) | Available (90) | Available (63) | 372 | 124 |
| Facility 9 | Available (38) | Available (25) | Available (644) | 707 | 235.7 |
| Facility 10 | Available (60) | - | - | 60 | 20 |
| Facility 11 | Available (53) | Available (233) | Available (11) | 297 | 99 |
| Facility 12 | Available (285) | Available (100) | Available (484) | 869 | 289.7 |
| Facility 13 | Available (499) | Available (103) | - | 602 | 200.7 |
| Facility 14 | Available (66) | Available (65) | Available (35) | 166 | 55.3 |
| Facility 15 | Available (67) | Available (383) | Available (192) | 642 | 214 |
| Facility 16 | Available (27) | Available (183) | Available (167) | 377 | 125.7 |
| Facility 17 | Not Available (0) | Available (433) | Available (56) | 489 | 163 |
| ***In Jigawa state*** |  |  |  |  |  |
| Facility 1 | Available (119) | Available (172) | - | 291 | 97 |
| Facility 2 | Available (111) | Available (77) | Available (178) | 366 | 122 |
| Facility 3 | Available (25) | Available (67) | - | 92 | 30.7 |
| Facility 4 | Available (239) | Available (196) | - | 435 | 145 |
| Facility 5 | Available (57) | Available (432) | Available (85) | 574 | 191.3 |
| Facility 6 | Available (75) | Available (190) | Available (60) | 325 | 108.3 |
| Facility 7 | Available (234) | Available (283) | Available (334) | 851 | 283.7 |
| Facility 8 | Available (388) | Available (80) | Available (595) | 1,063 | 354.3 |
| Facility 9 | Available (56) | Available (36) | Available (233) | 325 | 108.3 |
| Facility 10 | Available (523) | Available (169) | Available (280) | 972 | 324 |
| Facility 11 | Available (50) | Available (298) | Available (124) | 472 | 157.3 |
| ***In Kano state*** |  |  |  |  |  |
| Facility 1 | Available (60) | - | - | 60 | 20 |
| Facility 2 | Available (44) | - | - | 44 | 14.3 |
| Facility 3 | Available (209) | Available (179) | - | 388 | 129.3 |
| Facility 4 | Available (97) | - | - | 98 | 32.3 |
| ***In Lagos state*** |  |  |  |  |  |
| Facility 1 | Available (317) | - | - | 317 | 105.7 |
| Facility 2 | Available (0) | Available (152) | Available (258) | 536 | 178.7 |
| Facility 3 | Available (49) | - | - | 49 | 16.3 |
| Facility 4 | Available (117) | Available (184) | Available (45) | 346 | 115.3 |
| Facility 7 | Available (389) | Available (80) | - | 469 | 156.3 |
| Facility 6 | Available (25) | - | - | 25 | 8.3 |
| Facility 7 | Available (294) | Available (120) | - | 414 | 138 |
| Facility 8 | Available (170) | - | - | 170 | 56.7 |
| ***In Rivers state*** |  |  |  |  |  |
| Facility 1 | Available (240) | Available (135) | - | 375 | 202.7 |
| Facility 2 | Available (22) | - | - | 22 | 7.3 |
| Facility 3 | Available (328) | Available (265) | - | 593 | 197.7 |
| Facility 4 | Available (103) | - | - | 103 | 34.3 |
| Facility 5 | Available (330) | - | - | 330 | 110 |
